# Circulating endothelial extracellular vesicle signatures correspond with ICU requirement: an exploratory study in COVID-19 patients

**DOI:** 10.1186/s40635-023-00567-7

**Published:** 2023-11-30

**Authors:** Johannes Zipperle, Johannes Oesterreicher, Matthias Hackl, Teresa Lara Krammer, Helena Thumfart, Madhusudhan Reddy Bobbili, Marion Wiegele, Johannes Grillari, Marcin F. Osuchowski, Herbert Schöchl, Wolfgang Holnthoner, Christoph J. Schlimp, Judith Schiefer, Marco Valerio Pesce, Stefan Ulbing, Johannes Gratz

**Affiliations:** 1 Ludwig Boltzmann Institute for Traumatology, The Research Center in Cooperation With AUVA, Vienna, Austria; 2 Austrian Cluster for Tissue Regeneration, Vienna, Austria; 3 TAmiRNA GmbH, Vienna, Austria; 4 Institute for Molecular Biotechnology, Department for Biotechnology, University of Natural Resources and Life Sciences, Vienna, Austria; 5 Department of Anaesthesia, Intensive Care Medicine and Pain Medicine, Medical University of Vienna, Vienna, Austria; 6 AUVA Trauma Center Salzburg, Department of Anaesthesiology and Intensive Care Medicine, Academic Teaching Hospital of the Paracelsus Medical University, Salzburg, Austria; 7 Department of Anaesthesiology and Intensive Care Medicine, AUVA Trauma Center Linz, Linz, Austria; 8 Ludwig Boltzmann Institute Digital Health and Patient Safety, Vienna, Austria

**Keywords:** Extracellular vesicles, Intensive care unit, Biomarker, miRNAs, COVID-19, SARS-CoV-2, Critical care medicine

## Abstract

Extracellular vesicles (EVs) represent nanometer-sized, subcellular spheres, that are released from almost any cell type and carry a wide variety of biologically relevant cargo. In severe cases of coronavirus disease 2019 (COVID-19) and other states of systemic pro-inflammatory activation, EVs, and their cargo can serve as conveyors and indicators for disease severity and progression. This information may help distinguish individuals with a less severe manifestation of the disease from patients who exhibit severe acute respiratory distress syndrome (ARDS) and require intensive care measures. Here, we investigated the potential of EVs and associated miRNAs to distinguish normal ward patients from intensive care unit (ICU) patients (*N* = 10/group), with 10 healthy donors serving as the control group. Blood samples from which plasma and subsequently EVs were harvested by differential ultracentrifugation (UC) were obtained at several points in time throughout treatment. EV-enriched fractions were characterized by flow cytometry (FC), nanoparticle tracking analysis (NTA), and qPCR to determine the presence of selected miRNAs. Circulating EVs showed specific protein signatures associated with endothelial and platelet origin over the course of the treatment. Additionally, significantly higher overall EV quantities corresponded with increased COVID-19 severity. MiR-223-3p, miR-191-5p, and miR-126-3p exhibited higher relative expression in the ICU group. Furthermore, EVs presenting endothelial-like protein signatures and the associated miR-126-3p showed the highest area under the curve in terms of receiver operating characteristics regarding the requirement for ICU treatment. In this exploratory investigation, we report that specific circulating EVs and miRNAs appear at higher levels in COVID-19 patients, especially when critical care measures are indicated. Our data suggest that endothelial-like EVs and associated miRNAs likely represent targets for future laboratory assays and may aid in clinical decision-making in COVID-19.

**Supplementary Information:**

The online version contains supplementary material available at 10.1186/s40635-023-00567-7.

## Introduction

Coronavirus disease 2019 (COVID-19) is caused by a newly emerged member of the *coronaviridae* family: severe acute respiratory syndrome coronavirus-2 (SARS-CoV-2), first described at the end of 2019 in the Hubei province of China. COVID-19 has since affected more than 500 million people worldwide, with a global death toll of over six million [1]. SARS-CoV-2 infection is associated with a dysregulated immuno-inflammatory response, a dysfunctional endothelium, and robust pro-thrombotic activation [2, 3]. Extracellular vesicles (EVs) have emerged as an interesting investigative target in the pathophysiology of COVID-19. These nanometer-sized vesicles have been studied both as diagnostic biomarkers and as components of potential treatment strategies [4]. EVs are released from nearly all cell types and contribute to intercellular signal transduction in health and disease. Based on their general role in transmitting information and bioactive material between cells, EVs have been studied as potential drivers and biomarkers in COVID-19 [5]. Variations of circulating EVs (in terms of their number and activity) in the blood have been linked to the disease [6, 7]. In addition to the detection of quantitative changes and the assessment of cellular origin based on membrane composition, EV-associated cargo molecules like microRNAs (miRNAs) represent targets that are capable of modulating the expression of genes involved in thrombosis and inflammation. In this context, miRNAs have been studied extensively and established as diagnostic markers with a potentially broad range of health implications [8, 9]. MiRNA genes are transcribed by RNA polymerase II and are considered non-coding RNAs that are not translated into peptide sequences. The accessibility of miRNAs through most body fluids, combined with their specificity throughout disease progression, has been fundamental for their use in clinical settings [10]. With regard to COVID-19, direct and indirect cellular responses to the infection have gain considerable importance in the understanding of disease progression and severity. In SARS-CoV-2 infections, the epithelium of the upper and lower respiratory tract is considered the main entry (and replication) point of the virus, primarily mediated by an expression of angiotensin-converting enzyme 2 (ACE2) and transmembrane protease serine subtype 2 (TMPRSS2) on the cell surface. Interestingly, the late SARS-CoV-2 variants developed an ability to enter the cells in a TMPRSS2-and/or ACE2-independent manner enabling infection of a wider range of cells [11–13]. Regarding the role of the endothelium in COVID-19 pathophysiology, the most recent data propose that SARS-CoV-2 spike protein binds to endothelial cells by interacting with different host receptors and leads to various types of endothelial injury [14]. Regardless of the precise mechanistic involvement, endothelium constitutes an important interface for the most severe COVID-19 phenotypes including endotheliitis [3, 15]. This extensive interaction of SARS-CoV-2 with the host’s vasculature underscores the importance of biomarkers indicative of endothelial health and disease [16]. The combination of (endothelial) EVs and associated miRNAs as signatures brought forth a new aspect of both entities with regard to their potential as markers and therapeutics in pathophysiological conditions [17].

In this study, we aim to explore circulating EVs and their possible cellular origins in the bloodstream of COVID-19 patients. By combining the expression signatures of three preselected target miRNAs and defined EV populations, we explored the association with ICU treatment requirement in a small cohort of COVID-19 patients. Using flow cytometry, nanoparticle tracking analysis, and qPCR, we intend to gain insight into variations in circulating EV-associated signatures, ideally linking them to COVID-19 severity and progression. Our exploratory investigation aims to advance the understanding of this complex disease and to identify targets for the establishment of biomarkers in future studies and diagnostic platforms.

## Materials and methods

### Study design

This observational cohort study was conducted at the Medical University of Vienna, the Ludwig Boltzmann Institute for Traumatology and TAmiRNA GmbH, all of which are located in Vienna, Austria. It included patients afflicted with COVID-19 from three intensive care units (Department of Anaesthesia, Intensive Care Medicine and Pain Medicine) and one regular ward (Department of Medicine I, Division of Infectious Diseases and Tropical Medicine) at the Medical University Hospital of Vienna. Healthy volunteers acted as the control group. This investigation was approved by the local ethics committee of the Medical University of Vienna (vote nr. EK 1590/2020) and was carried out in accordance with the Declaration of Helsinki. Furthermore, all applicable municipal, state, and federal legislation were complied with throughout the study. Written informed consent was obtained from all patients who were able to consent. For patients who were unable to consent and who succumbed during the course of intensive care treatment, the ethics committee waived the need for informed consent. The study was designed, data was handled, and results were reported in line with the STROBE guidelines, ensuring the highest standards in research quality. A consecutive cohort of adults (≥ 18 years) who exhibited COVID-19 symptoms and tested positive for the SARS-CoV-2 virus via PCR at the time of hospitalization were enrolled in the study. Patients with moderate COVID-19 symptoms and requirement for oxygen administration were admitted to the regular ward, while those with severe ARDS were admitted to the ICU. As the Medical University of Vienna Hospital is a tertiary care center, most ICU patients were transferred from other critical care facilities to evaluate the indication for ECMO therapy. The course of treatment adhered to international guidelines for the care of COVID-19 [18, 19]. All patients were treated with enoxaparin as an anticoagulant throughout the course of the study. To obtain demographic and medical data from patients in the regular ward, information was extracted from the central patient data management system (SAP NetWeaver^®^7.0, SP Stack 17, including BI Content Add-On 3, SP09, September 2008, SAP AG). Patient data from the ICU were automatically documented via the IntelliSpace Critical Care and Anaesthesia Patient Data Management System (ICCA,Philips GmbH, Healthcare, Amsterdam, Netherlands). Blood samples were collected from both ICU and normal ward patients at five separate occasions: admission (T1), day 1 (T2), day 3 (T3), day 5 (T4), and day 7 (T5). For patients in the regular ward, samples were taken during routine blood sampling. Blood samples from ICU patients were taken from an arterial line or through central venous access. Healthy volunteers in the control group underwent a single venipuncture to obtain their study samples. Plasma samples were obtained by centrifugation and frozen at − 80 °C until further processing.

### Enrichment of EVs by differential centrifugation

The enrichment of EVs was performed by subjecting patient and volunteer plasma samples to differential centrifugation steps. Since our study focused on differences in absolute counts, our experimental protocol was optimized to keep the volume of plasma which was subjected to enrichment at 100 µL. This volume was maintained in all conditions and at all time points to obtain consistent information about quantitative differences. After thawing the vials in a water bath, the tubes were immediately put on ice. In order to clear the specimens from debris and large conglomerates, the tubes were centrifuged at 1500×*g* for 10 min, and the supernatant plasma was subsequently transferred to ultracentrifugation tubes (Ultra-Clear, Beckmann Coulter, Carlsbad, CA, USA). Samples were diluted with 12.5 mL of cold, sterile-filtered PBS 1 × (without Ca^++^ Mg^++^) prior to centrifugation. Ultracentrifugation was performed on a swing out rotor (SW40.1 Ti) in a L-100XP Ultracentrifuge (Beckmann Coulter, Carlsbad, CA, USA) at 100,000×*g* for 65 min (including acceleration time) at 4 °C in a vacuum. Whereas the supernatant liquid fraction was carefully discarded, the pellet at the tube bottom was thoroughly resuspended in cold, sterile-filtered PBS 1 × (without Ca^2+^ Mg^2+^). In every sample, the amount of PBS used for resuspension was equivalent to the volume of plasma that had been brought into processing (100 μL). All samples were kept on ice and frozen in 1.5 mL tubes (DNA LoBind, Eppendorf, Hamburg, Germany) at − 80 °C immediately until further downstream analysis.

### Flow cytometric analysis of EVs

The flow cytometry of EV-enriched samples was performed using fluorescence triggering combined with a multicolor fluorophore panel [20]. In brief, after the samples were thawed and vortexed, 80 μL of sample was transferred to a round-bottom flow cytometry tube and kept on ice in the dark throughout the entire procedure. To differentiate vesicles from protein aggregates and debris, lipid bilayers were stained with the membrane-anchoring dye CellMask™ green (CMG, Invitrogen, Waltham, MA, USA) to be detected on the flow cytometer’s FITC filterset. A total of 5 μL of CMG (diluted 1:5000 in sterile-filtered PBS [without Ca^2+^ Mg^2+^]) was added per tube. To identify endothelial-derived EVs, the samples were co-stained with a PE-labeled monoclonal antibody against platelet endothelial cell adhesion molecule-1 (mouse anti-human CD31, PECAM-1, clone WM59; BD Life Sciences, Franklin Lakes, NJ, USA), platelet glycoprotein Ib alpha chain (anti-human CD42b, GPIb, monoclonal antibody, APC-eFluor™ 780, eBioscience™, San Diego, CA, USA), monocyte/macrophage TLR receptor 2 (mouse anti-human CD14 monoclonal antibody, clone 61D3, PE-Cyanine7, eBioscience™), transmembrane tetraspanin (mouse anti-human CD81 monoclonal antibody, clone 1D6-CD81, PerCP-eFluor™ 710, eBioscience™) and tissue factor (mouse anti-human CD142 monoclonal antibody, clone HTF-1, APC, eBioscience™) at a volume of 3 µL each. All antibody solutions were spun down at 16,000×*g* for 10 min prior to staining. After vortexing, the samples were incubated at 37 °C for 30 min in darkness. In order to dilute unbound dye and antibodies, 200 μL of cold, sterile-filtered PBS 1 × (without Ca^2+^ Mg^2+^) were added. Samples were put on ice immediately and kept in the dark until measurement. Flow cytometric analysis was performed using a Cytoflex cytometer (Beckman Coulter, Brea, CA, USA). Single and fluorescence minus one (FMO) stainings were performed for compensation and to correct potential fluorophore spillover. Gating was based on the positivity of events for CMG (phospholipid bilayer) to detect EVs and other membranous particles. Detergent controls in the presence of standard RIPA lysis and extraction buffer were performed to confirm the specificity of the CMG signal for phospholipid bilayers. CMG + events were further evaluated for the presence of cell-specific antigens and are denoted in absolute counts as events/μL. As an additional marker for quality control, events were assessed for the positivity for the tetraspanin CD81. This transmembrane protein is one of three tetraspanins (CD9, CD63 and CD81) to be most abundant on EVs enriched from human plasma by ultracentrifugation [21]. A comprehensive overview over the establishment procedure of the flow cytometry protocol is given in Additional file 1: Fig. S1. The recorded measurements were analyzed using the software CytExpert 1.2 (Beckman Coulter, Brea, CA, USA).

### NTA measurements of EV samples derived from patient plasma

For the assessment of potential differences in particle concentration and size among different groups, nanoparticle tracking analysis (NTA) was performed with an Zetaview^®^ Quatt (ParticleMetrix, Inning am Ammersee, Germany). Enriched EV samples were diluted 1:150 to a total volume of 1500 μL for each measurement with PBS (w/o Ca^2+^/Mg^2+^) which was freshly filtered using a 0.22 μm PVDF syringe filter (Carl Roth, Karlsruhe, Germany). Measurements for all samples were performed in scatter mode with the device set to 11 positions, sensitivity at 80, shutter at 80, frame rate at 30, minimal brightness at 20, a minimum area of 10, a maximum area of 1000, and a trace length of 15. The device was calibrated, and performance was checked each day of measurement with standard beads of known size provided by the device supplier. Daily performance checks were considered successful when the trueness and precision values did not exceed 0.9% of 100 nm.

### RNA extraction and qPCR analysis

The number of total RNA, including small RNAs, was isolated from 90 μL extracellular vesicles using the miRNeasy Mini Kit (Qiagen, Hilden, Germany). The samples were thawed at room temperature and diluted to 200 μL with nuclease-free water. For homogenization, 1000 μL Qiazol were added. Samples were mixed vigorously and incubated at room temperature for 10 min. 200 μL chloroform were added, samples were mixed again and incubated at ambient temperature for 3 min. For phase separation, samples were centrifuged at 12,000×*g* for 15 min at 4 °C. 650 μL aqueous phase was transferred to fresh tubes, and glycogen was added for enhanced precipitation. Binding to RNeasy Mini spin columns and washing steps were executed via a QIAcube liquid-handling robot. RNA was eluted in 30 μL nuclease-free water and stored at − 80 °C until further processing. Reverse transcription was carried out with the miRCURY LNA RT Kit (Qiagen) in accordance with the manufacturer’s instructions. 2 μL RNA were input per 10 μL reaction. Samples were then incubated at 42 °C for one hour followed by 95 °C for 5 min (heat inactivation). For miRNA quantification, qPCR analysis with the miRCURY SYBR Green Master Mix (Qiagen) and commercially available LNA-enhanced miRNA assays (Qiagen) was carried out. The final dilution of cDNA was 1:100. In this setup, only mature miRNA sequences were detected. To ensure the quality of the generated data, synthetic spike-ins (Qiagen) were added in equimolar amounts before RNA isolation (UniSp4) and reverse transcription (cel-miR-39-3p). qPCRs were carried out on a LightCycler 96 (Roche, Basel, Switzerland) with the following settings: 95 °C for 120 s (activation) and 45 cycles at 95 °C for 10 s and at 56 °C for 60 s. Melting curve analysis was performed by continuous acquisition between 55 °C and 98 °C. Cq values were calculated with a combination of the 2nd derivative maximum and the fits point method (LC96 Roche v1.1). The RNA spike-in (UniSp4) served as the normalization control [33].

### Statistical analysis

Data were collected in a Microsoft Office Excel spreadsheet (Microsoft Inc., Redmond, WA, USA). All statistical calculations were performed using GraphPad Prism 8.0 (GraphPad Software, La Jolla, CA, USA). Normal distribution was assessed using a Kolmogorov–Smirnov test. Depending on the distribution, one-way analyses of variance or Kruskal–Wallis tests were performed to compare selected pairs of data columns. Continuous variables in patient characteristics were expressed as medians with interquartile ranges in data tables. Spearman correlations were employed to calculate the correlation coefficients. Depending on r, correlations were considered weak (*r* = 0.20–0.39), intermediate (*r* = 0.40–0.59), strong (*r* = 0.60–0.79), or very strong (*r* ≥ 0.80). Receiver operating characteristic curves (ROC) were calculated to assess assignment to the ICU group at T1. The level of statistical significance was set at *p* < 0.05.

## Results

Between November 2020 and January 2021, 30 individuals were included in the study. Twenty patients who tested positive for a SARS-CoV-2 infection were treated at either a normal ward or an ICU (*n* = 10 each), depending on the severity of the condition. Ten healthy donors served as the control group. An overview of patient and donor baseline characteristics is given in Table 1. As to the ICU patients, six patients (60%) underwent ECMO treatment, five patients (50%) underwent prone positioning, two patients (20%) underwent renal replacement therapy, and seven patients (70%) received catecholamines. Eight ICU patients (80%) eventually succumbed, whereas all normal ward patients included in the study survived throughout the study period.

**Table 1:** Baseline patient characteristics

	Unit	Healthy	Normal ward	ICU
Sex. *f* (%)		3 (30)	3 (30)	3 (30)
Age. Median (IQR)	Years	44 (40.5–46.25)	64 (46–78)	60.5 (52.25–67.5)
BMI. Median (IQR)		n/a	26 (23.8–35.65)	27.95 (25.13–40.6)
Hb. Median (IQR)	g/dL	14.9 (13.68–15.33)	12.75 (12.15–14.35)	10.05 (8.52–11.23)
Plt. Median (IQR)	G/L	241.5 (206.5–286)	200 (119–232.5)	195.5 (129–253)
LEU	G/L	6.14 (4.92–6.96)	5.76 (4.59–6.28)	11.34 (5.47–14.48)
CRP	mg/dL	0.06 (0.04–0.13)	5.98 (1.47–12.21)	24.53 (13.83–32.67)

### COVID-19 causes the release of EVs from differential cellular origins

Several cell-specific antigen combinations were identified in the blood of the observed COVID-19 patients. With reference to vehicle controls and the optimization of a cell-marker green (CMG)-positive signal, phospholipid bilayer-positive events were identified as EVs.

**Fig. 1:**
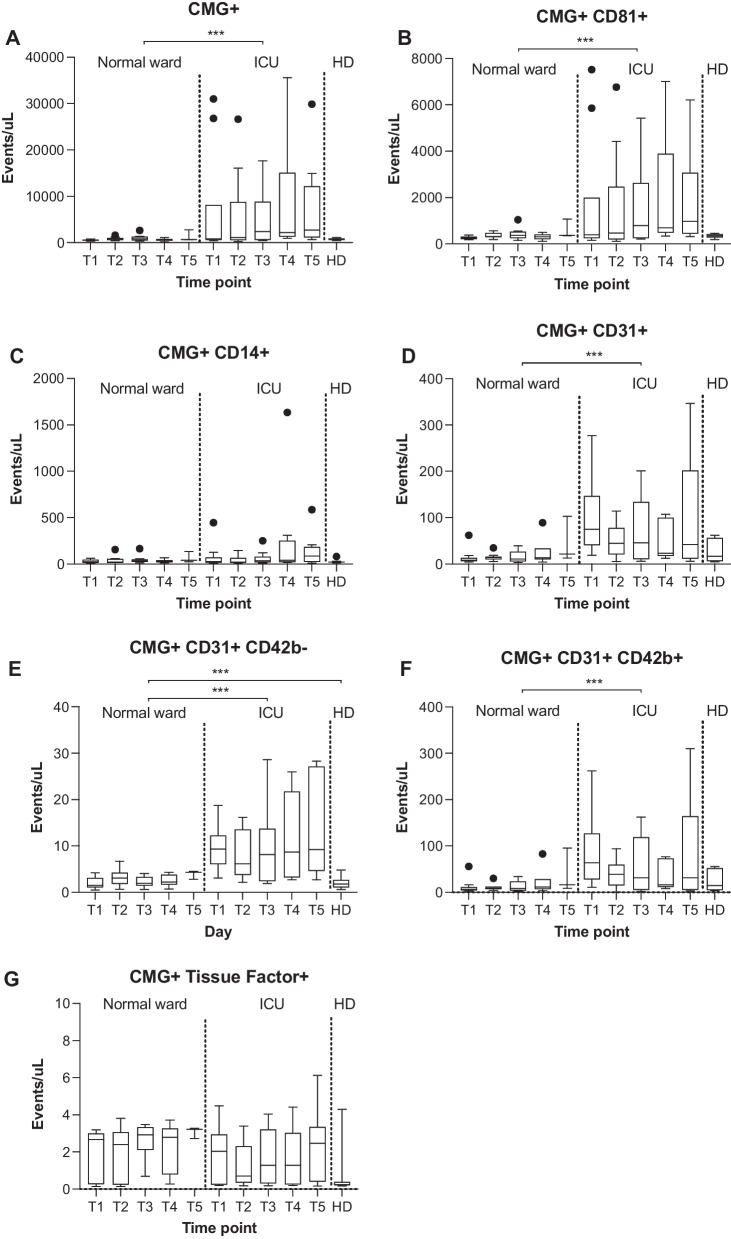
Absolute counts of extracellular vesicles from different cellular origins in the circulation of normal ward and ICU patients throughout the observation period. Data are shown as box and whisker blots with Tukey depictions of outliers. One-way analyses of variance or Kruskal–Wallis tests were performed to compare pooled time points in normal ward patients, ICU patients, and healthy donors (HD). If not indicated otherwise by lines, differences in groupwise comparisons were non-significant. *p < 0.05; **p < 0.01, ***p < 0.001

There was a significantly higher absolute count of EVs present in ICU patients when compared to patients on the normal ward (Fig. 1A).

CMG positivity was used as a prerequisite for further gating and the identification of antigen patterns indicative of the cellular origin of EVs. A significantly higher concentration of EVs rendered positive for tetraspanin CD81 in ICU patients compared to normal ward patients (Fig. 1B). A monocyte- and macrophage-derived EV population was detected based on positivity for CMG and the presence of the lipopolysaccharide receptor CD14. There was no difference in the EV counts in this population, including the healthy controls (Fig. 1C).

A measurement of platelet and endothelial cell adhesion molecules on EVs (CMG+CD31+) revealed significantly higher counts in the ICU group (Fig. 1D). Upon the subtraction of the positivity for platelet CD42b, a likely endothelial-like population of EVs was identified—this population was more abundant in the ICU patients (Fig. 1E). The platelet-specific population was larger in ICU patients than in normal ward patients (Fig. 1F). There was no difference between normal ward and ICU patients in tissue factor-positive EVs (Fig. 1G).

### COVID-19 patients reveal higher quantities of larger EVs

**Fig. 2:**
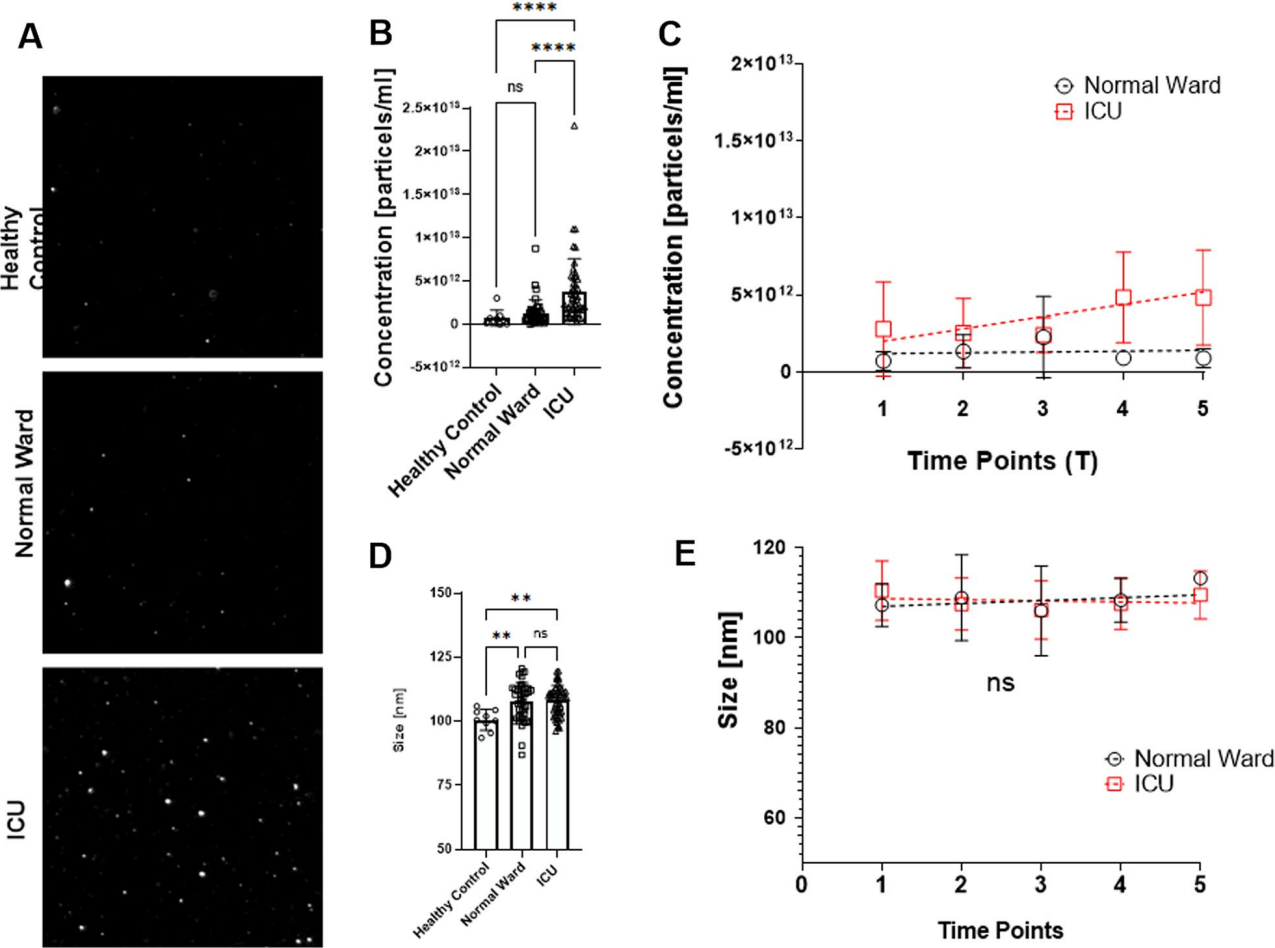
**A** Representative results of nanoparticle tracking analysis **B**, **C** Cumulative and time-dependent concentration of particles. **D**, **E** Cumulative and time-dependent average diameter of particles. One-way analyses of variance or Kruskal–Wallis tests were performed to compare time points or data from pooled sampling points. *p < 0.05; **p < 0.01, ***p < 0.001, *ns* non-significant

Results from label-free nanoparticle tracking analysis (NTA) of samples in the observed healthy donors, normal ward patients, and ICU patients are depicted in Fig. 2. Representative screenshots from selected measurements indicated a higher particle count in the ICU patients (Fig. 2A). Quantitative results of the tracking analysis over all time points revealed higher overall concentrations in the ICU group compared to normal ward patients and healthy controls (Fig. 2B). However, there were no significant differences between the trajectories of the normal ward and ICU patients regarding particle concentrations over time (Fig. 2C). A cumulative comparison of average particle size demonstrated that particles were significantly larger in COVID-19 patients than in healthy donors, whereas we found no difference between the ICU and the normal ward groups (Fig. 2D). Additionally, there was no time-dependent difference in size between the normal ward patients and the ICU patients (Fig. 2E).

### Elevated levels of specific miRNAs are associated with ICU requirement

**Fig. 3:**
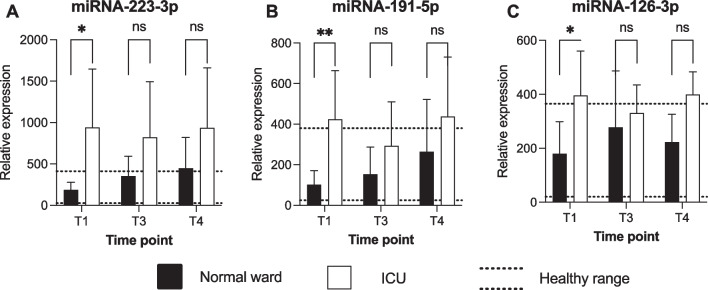
Relative expression of selected miRNAs in the circulation of normal ward and ICU patients. Expression of each target was normalized to the median level of this respective miRNA in the healthy donor group and is given as percentage. The dotted lines illustrate the range of healthy donors. One-way analyses of variance or Kruskal–Wallis tests were performed to compare data from different time points. *p < 0.05; **p < 0.01, ***p < 0.001; *ns* non-significant

Next, we analyzed the levels of the three miRNAs at different time points throughout the observation period. There was a higher expression of miR-223-3p at T1 when comparing normal ward patients with ICU patients (Fig. 3A). There was a higher expression of miR-191-5p at T1 when comparing normal ward patients with ICU patients (Fig. 3B). In a similar fashion, miR-126-3p exhibited a higher expression at T1 in ICU patients in comparison with normal ward patients (Fig. 3C).

### Circulating endothelial-like EVs are associated with elevated levels of specific miRNAs

**Fig. 4:**
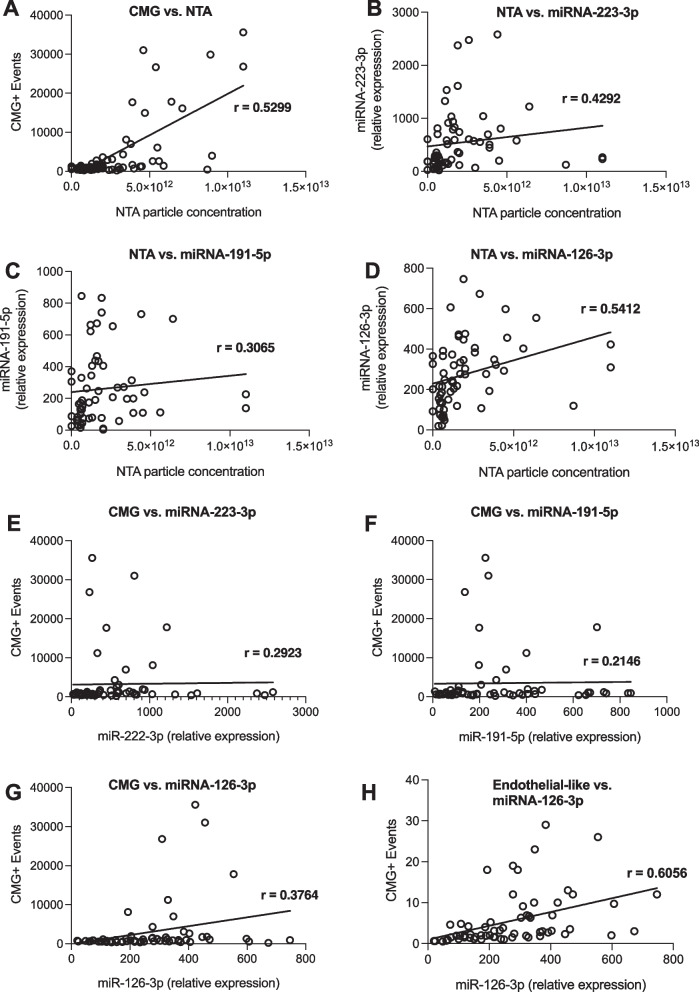
Spearman correlation of flow cytometry, NTA, and selected miRNA expression data in the circulation of normal ward and ICU patients. *CMG* cell mask green, *NTA* nanoparticle tracking analysis

To investigate the potential correlation between EVs and the selected target miRNAs, we analyzed results across assays over the entire dataset. A quantitative comparison between flow cytometry and NTA showed an intermediate correlation between the two technologies (Fig. 4A).

To further investigate indicators for the potential co-localization of miRNAs with EVs, particle counts in the NTA were plotted against all three analyzed miRNAs. All three miRNAs showed a weak to intermediate correlation between NTA particle concentrations and the relative expression of the targets (Fig. 4B–D). In order to substantiate these findings on another platform, the expression of the three miRNAs was correlated with CMG+event count in flow cytometry. There was a weak correlation between CMG+EV concentrations and the relative expression of miR-223-3p and miR-191-5p in the samples (Fig. 4E, F). However, similar to the NTA findings, there was a higher correlation between CMG+events and miR-126-3p expression (Fig. 4G). Gating the flow cytometry results for specific cellular origins, the correlation of miR-126-3p expression with EV count could be substantially increased when focusing on the endothelial-like EV population (Fig. 4H).

### Endothelial EVs and miRNAs are associated with ICU requirement in COVID-19 patients

**Fig. 5:**
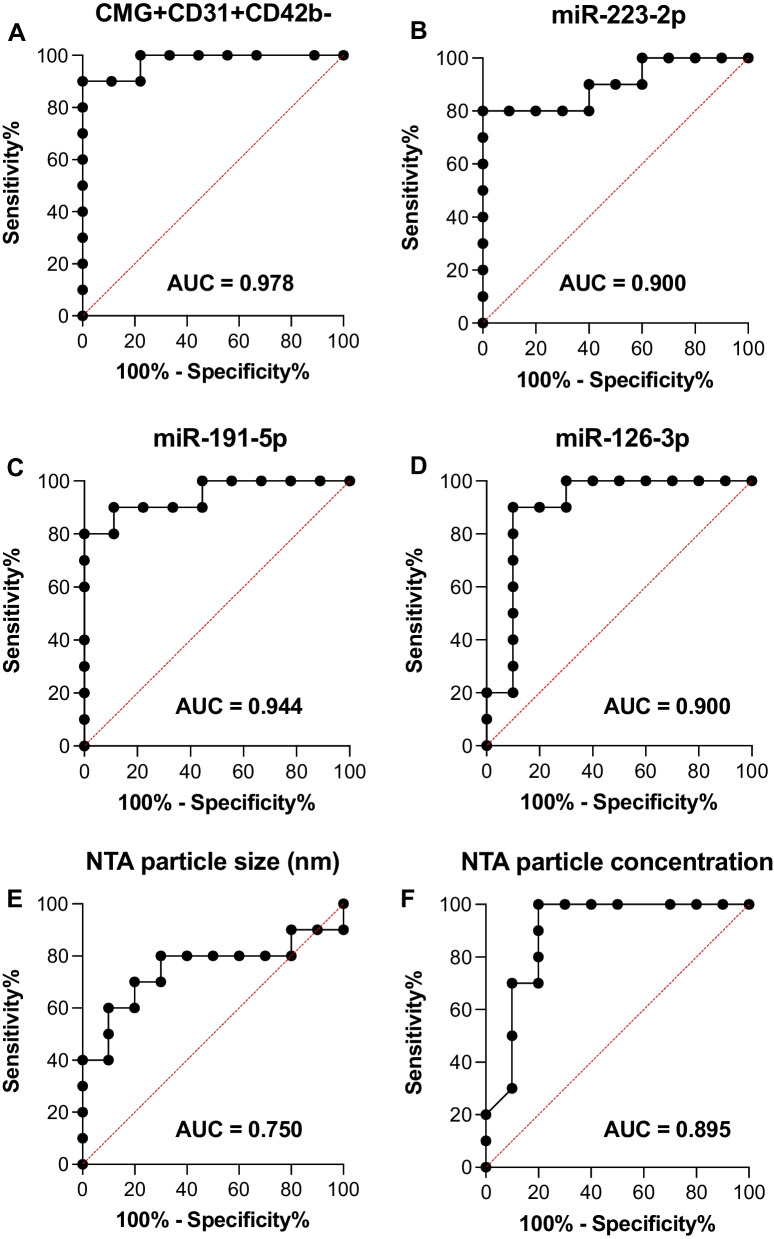
Receiver operating characteristics (ROC) of flow cytometry, NTA, and miRNA expression data regarding assignment to the ICU group at T1

Given that many of the investigated parameters consistently showed differences at admission, we consequently investigated the specificity and sensitivity of those markers to predict assignment to the ICU group at T1. The endothelial EV population (CMG + CD31 + CD42b−) showed the highest association with ICU requirement (Fig. 5A). All three assessed miRNAs were associated with an assignment to the ICU group, but differed in accuracy (Fig. 5B–D). Similarly, particle concentration in the NTA showed discriminatory potential, whereas particle size had a weaker predictive accuracy (Fig. 5E, F).

## Discussion

Like other systemic viral infections, COVID-19 is associated with a dysregulated inflammatory response, dysfunctional endothelium, and prothrombotic activation [2, 3]. The endothelium and its vesicular content play an important role in the onset of the disease, thus representing promising candidates for diagnosis, therapy, and drug delivery. In this exploratory study, we compared miRNAs and EVs from different cellular origins in healthy volunteers, patients with COVID-19 in a normal ward, and critically ill patients with COVID-19. Currently, various laboratory parameters have been associated with COVID-19 disease severity and the need for ICU admission [22–25]. In this study, we investigated the potential of EVs and associated cargo to serve as complementary biomarker candidates in addition to the established panel of clinical laboratory parameters. Our flow cytometric assessment of EVs from different tissues revealed that the EV population showing endothelial-like antigen patterns, identified by positivity for CMG (phospholipid bilayer), CD31 (platelet–endothelial cell adhesion molecule-1, PECAM-1), and negativity for CD42b (platelet glycoprotein Ib alpha chain), showed a favorable signal-to-noise-ratio, with regard to the difference in ICU and normal ward patients. Although lacking cell-specific antigens to identify their origins, particle concentration in NTA correlated with flow cytometry results, confirming increased levels of EVs in the ICU group. Interestingly, NTA also suggests that EVs are larger in COVID-19 patients, which is in line with the nature of vesicles that shed from cell membranes in response to activation and/or apoptosis [26]. Depending on their diameter and biogenesis, EVs are mostly subdivided into exosomes, microvesicles, and apoptotic bodies [27, 28]. The larger portion of EVs, often termed microvesicles, has proven its potential as a danger signal in various conditions and has been shown to differentially affect the thrombotic micromilieu in affected patients and in ex vivo settings [29–31]. These characteristics render the microvesicle EV fraction a promising target for providing insights into disease progression along established, danger-associated molecular patterns (DAMPs). EVs serve as biological carrier systems for a number of membrane-bound and luminal molecules. Against this background, our investigation focused on established miRNAs with a known or suggested association with EVs to substantiate the concept of EV signatures, thereby combining membrane composition, cellular origin and specific cargo molecules. Specific profiles of EV-associated miRNAs have previously been reported, including in publicly available databases [32]. Indeed, in the present study, all miRNA targets (miR-223-3p, miR-191-5p, and miR-126-3p) showed a significantly higher expression in ICU patients at T1 when compared to normal ward patients and healthy individuals. All three miRNA targets appeared relevant with regard to severe infection since they have been previously reported to be enriched in endothelial cells, immune cells, and platelets [33–35]. Apart from miRNAs having a role in the host response to infection, plasma levels of all three targets have been shown to be responsive to changes in platelet function, which is often affected by the dysregulation of hemostasis [36]. In this context, miR-223 and miR-191 have been established as solid platelet miRNAs. MiR-223 is known to target P2Y12 receptor mRNA, thereby regulating platelet reactivity,furthermore, it is expressed in leukocytes, where it modulates inflammatory activation [37]. MiR-126 is highly enriched in ECs and is involved in endothelial barrier function and integrity [34]. Although these observations do not signify a causative relation, our assessment of the correlation of these miRNAs with the presence of EVs suggested an association of miR-126-3p with circulating vesicles, whereas miR-223-3p and miR-191-5p showed a weaker correlation. A further narrowing down of the association of miR-126-3p expression with EVs that showed endothelial-like antigen patterns substantially increased the correlation, suggesting that these targets displayed a spatial and temporal co-occurrence in the circulation of the assessed subjects. This finding is in line with previous studies reporting that miR-126 is endothelial-specific [38]. Furthermore, we suggest that increased endothelial-like EV signatures are associated with ICU requirement and potentially worse outcomes in COVID-19 patients. Due to the exploratory and observational nature of this study and its small sample size, we could not assess the actual predictability of clinical progression by measuring EVs and miRNAs over time. However, by comparing T1 in all groups, we calculated the sensitivity and specificity by which distinct parameters were associated with ICU requirement. To quantify the predictive power of a target with regard to ICU requirement, we referred to the area under the curve (AUC) for comparison. The ability of the employed cytometer to provide a semi-quantitative assessment of the analyzed EVs in events/μL facilitated the calculation of cut-off values based on the Youden index (data not shown). Upon comparison of AUCs, the endothelial EV population (CMG+CD31+CD42b−) showed the highest predictive power, followed by the three assessed miRNAs. Although still of diagnostic significance, NTA particle size and concentration showed less discriminatory accuracy with regard to ICU requirement. In contrast to NTA, flow cytometry can specifically identify EVs from different cellular origins. This substantiated our hypothesis that the endothelial EV fraction was not only an essential pro-inflammatory indicator of cellular activation but might represent a promising target for theragnostic approaches. To face the challenge of requiring highly sophisticated methods to detect EVs and their associated cargo like miRNAs, platforms to quantify these targets in a rapid fashion, including at the point of care, remain the subject of current research efforts [39–42].

### Limitations

Although this exploratory investigation provides valuable insights into the potential of EV-associated targets to serve as biomarkers, it is limited by the observational study design, the small sample size, and the lack of translatability into current clinical practice. Furthermore, patients for this study were recruited at a tertiary care center, primarily accepting patients with the requirement for ECMO support. Thus, it needs to be recognized that the ICU group of or study carries a bias towards the most severe manifestation of COVID-19. Although we considered a subgroup analysis for patients with and without ECMO support, we decided not to perform it due to the small sample size. In addition, the number of included patients prevented us from providing a more granular depiction and analysis of changes over time, which might be of particular interest during the dynamic process of COVID-19. Another limitation is the lack of temporal continuity, which prevents the application of actual prediction models. This circumstance has not only limited our ability to draw detailed conclusions, predictions, and deductions, but has also resulted in a lack of diversity in the data. Not least, this lack of diversity was reflected by the difference of age between the two patient cohorts and the control group. Furthermore, EV isolation (enrichment) and detection represent non-standardized procedures with a high magnitude of analytical variability and proneness to error. Although our experimental approach provides valuable insights in the abundance of certain EV subtypes, it does not depict the entireness of circulating populations including potential shifts in relative changes. Our findings substantiate the role of EVs and miRNAs as potentially important biomarkers and call for additional clinical research to be conducted in larger cohorts, ideally including highly standardized preparation and detection technology at the point of care.

## Conclusions

Our exploratory investigation of the diagnostic utility of EVs and miRNAs in COVID-19 revealed the emergence of higher quantities of circulating EVs from different origins in COVID-19 patients, particularly in those who require ICU treatment. Circulating EVs in COVID-19 patients appeared to be larger in diameter compared to those in the healthy control group, suggesting the release from the cell membrane in response to activation. Endothelial EVs appeared to exhibit a favorable signal-to-noise ratio with regard to the differentiation between normal ward and ICU patients. This was also manifested in a higher relative r when endothelial EVs were correlated with miRNAs with an increased specificity for endothelial cells. Both endothelial EVs and associated miRNAs showed an association with ICU requirement. Our findings suggest that endothelial EVs and associated miRNAs might represent promising theragnostic candidates for patients with COVID-19.

### Supplementary Information

**Additional file 1: Fig. S1**. Gating strategy in the flow cytometric analysis of the study cohort.

## Data Availability

All data analysed in the current study is available from the corresponding author upon reasonable request.

## References

[1] COVID-19 Dashboard by the Center for Systems Science and Engineering (CSSE) at Johns Hopkins University (JHU). ArcGIS. Johns Hopkins University. Retrieved 11 June 2022. 10.1016/S1473-3099(22)00434-0 PMC9432867 36057267

[2] Bernard I, Limonta D, Mahal LK, Hobman TC (2020). Endothelium infection and dysregulation by SARS-CoV-2: evidence and Caveats in COVID-19. Viruses.

[3] Osuchowski MF, Winkler MS, Skirecki T, Cajander S, Shankar-Hari M, Lachmann G, Monneret G, Venet F, Bauer M, Brunkhorst FM, Weis S, Garcia-Salido A, Kox M, Cavaillon JM, Uhle F, Weigand MA, Flohé SB, Wiersinga WJ, Almansa R, de la Fuente A, Martin-Loeches I, Meisel C, Spinetti T, Schefold JC, Cilloniz C, Torres A, Giamarellos-Bourboulis EJ, Ferrer R, Girardis M, Cossarizza A, Netea MG, van der Poll T, Bermejo-Martín JF, Rubio I (2021). The COVID-19 puzzle: deciphering pathophysiology and phenotypes of a new disease entity. Lancet Respir Med.

[4] Xie F, Su P, Pan T, Zhou X, Li H, Huang H, Wang A, Wang F, Huang J, Yan H, Zeng L, Zhang L, Zhou F (2021). Engineering extracellular vesicles enriched with palmitoylated ACE2 as COVID-19 therapy. Adv Mater.

[5] Hassanpour M, Rezaie J, Nouri M, Panahi Y (2020). The role of extracellular vesicles in COVID-19 virus infection. Infect Genet Evol.

[6] Guervilly C, Bonifay A, Burtey S, Sabatier F, Cauchois R, Abdili E, Arnaud L, Lano G, Pietri L, Robert T, Velier M, Papazian L, Albanese J, Kaplanski G, Dignat-George F, Lacroix R (2021). Dissemination of extreme levels of extracellular vesicles: tissue factor activity in patients with severe COVID-19. Blood Adv.

[7] Cappellano G, Raineri D, Rolla R, Giordano M, Puricelli C, Vilardo B, Manfredi M, Cantaluppi V, Sainaghi PP, Castello L, De Vita N, Scotti L, Vaschetto R, Dianzani U, Chiocchetti A (2021). Circulating platelet-derived extracellular vesicles are a hallmark of Sars-Cov-2 infection. Cells.

[8] Zhou SS, Jin JP, Wang JQ, Zhang ZG, Freedman JH, Zheng Y, Cai L (2018). miRNAS in cardiovascular diseases: potential biomarkers, therapeutic targets and challenges. Acta Pharmacol Sin.

[9] Bailey WJ, Glaab WE (2018). Accessible miRNAs as novel toxicity biomarkers. Int J Toxicol..

[10] Pogribny IP (2018). MicroRNAs as biomarkers for clinical studies. Exp Biol Med (Maywood).

[11] Baggen J, Jacquemyn M, Persoons L, Vanstreels E, Pye VE, Wrobel AG, Calvaresi V, Martin SR, Roustan C, Cronin NB, Reading E, Thibaut HJ, Vercruysse T, Maes P, De Smet F, Yee A, Nivitchanyong T, Roell M, Franco-Hernandez N, Rhinn H, Mamchak AA, Ah Young-Chapon M, Brown E, Cherepanov P, Daelemans D (2023). TMEM106B is a receptor mediating ACE2-independent SARS-CoV-2 cell entry. Cell.

[12] Alipoor SD, Mirsaeidi M (2022). SARS-CoV-2 cell entry beyond the ACE2 receptor. Mol Biol Rep.

[13] Willett BJ, Grove J, MacLean OA, Wilkie C, De Lorenzo G, Furnon W, Cantoni D, Scott S, Logan N, Ashraf S, Manali M, Szemiel A, Cowton V, Vink E, Harvey WT, Davis C, Asamaphan P, Smollett K, Tong L, Orton R, Hughes J, Holland P, Silva V, Pascall DJ, Puxty K, da Silva Filipe A, Yebra G, Shaaban S, Holden MTG, Pinto RM, Gunson R, Templeton K, Murcia PR, Patel AH, Klenerman P, Dunachie S, Haughney J, Robertson DL, Palmarini M, Ray S, Thomson EC, PITCH Consortium; COVID-19 Genomics UK (COG-UK) Consortium (2022). SARS-CoV-2 Omicron is an immune escape variant with an altered cell entry pathway. Nat Microbiol.

[14] Perico L, Benigni A, Remuzzi G. SARS-CoV-2 and the spike protein in endotheliopathy. Trends Microbiol. 2023. 10.1016/j.tim.2023.06.004 PMC10258582 37393180

[15] Varga Z, Flammer AJ, Steiger P, Haberecker M, Andermatt R, Zinkernagel AS, Mehra MR, Schuepbach RA, Ruschitzka F, Moch H (2020). Endothelial cell infection and endotheliitis in COVID-19. Lancet.

[16] Sardu C, Gambardella J, Morelli MB, Wang X, Marfella R, Santulli G (2020). Hypertension, thrombosis, kidney failure, and diabetes: is COVID-19 an endothelial disease? A comprehensive evaluation of clinical and basic evidence. J Clin Med.

[17] Mori MA, Ludwig RG, Garcia-Martin R, Brandão BB, Kahn CR (2019). Extracellular miRNAs: from biomarkers to mediators of physiology and disease. Cell Metab.

[18] Alhazzani W, Evans L, Alshamsi F (2021). Surviving sepsis campaign guidelines on the management of adults with coronavirus disease 2019 (COVID-19) in the ICU: first update. Crit Care Med.

[19] Alhazzani W, Møller MH, Arabi YM (2020). Surviving Sepsis Campaign: guidelines on the management of critically ill adults with Coronavirus Disease 2019 (COVID-19). Intensive Care Med.

[20] Oesterreicher J, Pultar M, Schneider J, Mühleder S, Zipperle J, Grillari J, Holnthoner W (2020). Fluorescence-based nanoparticle tracking analysis and flow cytometry for characterization of endothelial extracellular vesicle release. Int J Mol Sci.

[21] Paget D, Checa A, Zöhrer B, Heilig R, Shanmuganathan M, Dhaliwal R, Johnson E, Jørgensen MM, Bæk R, Wheelock CE, Channon KM, Fischer R, Anthony DC, Choudhury RP, Akbar N, Oxford Acute Myocardial Infarction Study (OxAMI) (2022). Comparative and integrated analysis of plasma extracellular vesicle isolation methods in healthy volunteers and patients following myocardial infarction. J Extracell Biol..

[22] Li X, Cao Y, Pan H (2020). Coagulation abnormalities in critically ill patients with COVID-19. J Thromb Haemost.

[23] Wu Z, McGoogan JM (2020). Characteristics of and important lessons from the Coronavirus disease 2019 (COVID-19) outbreak in China: summary of a report of 72314 cases from the Chinese Center for Disease Control and Prevention. JAMA.

[24] Ahmad T, Kabir AA, Fazal F (2020). Procalcitonin as a marker of severe lung injury in patients with severe COVID-19. J Clin Virol.

[25] Sun Y, Li Q, Xu W (2020). Clinical characteristics of severe and critical COVID-19 patients: a systematic review and meta-analysis. Int J Infect Dis.

[26] Kowal J, Tkach M, Théry C (2014). Biogenesis and secretion of exosomes. Curr Opin Cell Biol.

[27] Jeppesen DK, Fenix AM, Franklin JL, Higginbotham JN, Zhang Q, Zimmerman LJ, Liebler DC, Ping J, Liu Q, Evans R (2019). Reassessment of exosome composition. Cell.

[28] Buzas EI (2023). The roles of extracellular vesicles in the immune system. Nat Rev Immunol.

[29] Zipperle J, Schlimp CJ, Holnthoner W, Husa AM, Nürnberger S, Redl H, Schöchl H (2013). A novel coagulation assay incorporating adherent endothelial cells in thromboelastometry. Thromb Haemost.

[30] Holnthoner W, Bonstingl C, Hromada C, Muehleder S, Zipperle J, Stojkovic S, Redl H, Wojta J, Schöchl H, Grillari J, Weilner S, Schlimp CJ (2017). Endothelial cell-derived extracellular vesicles size-dependently exert procoagulant activity detected by thromboelastometry. Sci Rep.

[31] Raeven P, Zipperle J, Drechsler S (2018). Extracellular vesicles as markers and mediators in sepsis. Theranostics.

[32] Liu T, Zhang Q, Zhang J, Li C, Miao YR, Lei Q, Li Q, Guo AY (2019). EVmiRNA: a database of miRNA profiling in extracellular vesicles. Nucleic Acids Res.

[33] Zampetaki A, Willeit P, Tilling L, Drozdov I, Prokopi M, Renard JM, Mayr A, Weger S, Schett G, Shah A, Boulanger CM, Willeit J, Chowienczyk PJ, Kiechl S, Mayr M (2012). Prospective study on circulating MicroRNAs and risk of myocardial infarction. J Am Coll Cardiol.

[34] Sunderland N, Skroblin P, Barwari T, Huntley RP, Lu R, Joshi A, Lovering RC, Mayr M (2017). MicroRNA biomarkers and platelet reactivity: the clot thickens. Circ Res.

[35] Kaudewitz D, Skroblin P, Bender LH, Barwari T, Willeit P, Pechlaner R, Sunderland NP, Willeit K, Morton AC, Armstrong PC, Chan MV, Lu R, Yin X, Gracio F, Dudek K, Langley SR, Zampetaki A, de Rinaldis E, Ye S, Warner TD, Saxena A, Kiechl S, Storey RF, Mayr M (2016). Association of microRNAs and YRNAs with platelet function. Circ Res.

[36] Willeit P, Zampetaki A, Dudek K, Kaudewitz D, King A, Kirkby NS, Crosby-Nwaobi R, Prokopi M, Drozdov I, Langley SR, Sivaprasad S, Markus HS, Mitchell JA, Warner TD, Kiechl S, Mayr M (2013). Circulating microRNAs as novel biomarkers for platelet activation. Circ Res.

[37] Landry P, Plante I, Ouellet DL, Perron MP, Rousseau G, Provost P (2009). Existence of a microRNA pathway in anucleate platelets. Nat Struct Mol Biol.

[38] Wang S, Aurora AB, Johnson BA, Qi X, McAnally J, Hill JA, Richardson JA, Bassel-Duby R, Olson EN (2008). The endothelial-specific microRNA miR-126 governs vascular integrity and angiogenesis. Dev Cell..

[39] Xu J, Wang Y, Ma J (2020). Point-of-care diagnostics using extracellular vesicles. Biomater Sci.

[40] Groh F, Castillo D, Baek S (2020). Point-of-care diagnostics using extracellular vesicles from circulating tumors. Sensors.

[41] El-Khatib A, El-Khatib F, El-Khatib F (2020). Extracellular vesicles as diagnostics biomarkers in point-of-care applications. J Clin Med.

[42] Zampetaki A, Mayr M (2012). Analytical challenges and technical limitations in assessing circulating miRNAs. Thromb Haemost.

