# Supplementary material for: Circulating endothelial extracellular vesicle signatures correspond with ICU requirement: an exploratory study in COVID-19 patients
Source: Intensive Care Med Exp. 2023 Nov 30;11:85. doi: 10.1186/s40635-023-00567-7 (PMC10689640; doi:10.1186/s40635-023-00567-7)
Supplement: Supplementary file 1 — Additional file 1: Fig. S1. Gating strategy in the flow cytometric analysis of the study cohort. [file 40635_2023_567_MOESM1_ESM.pdf]

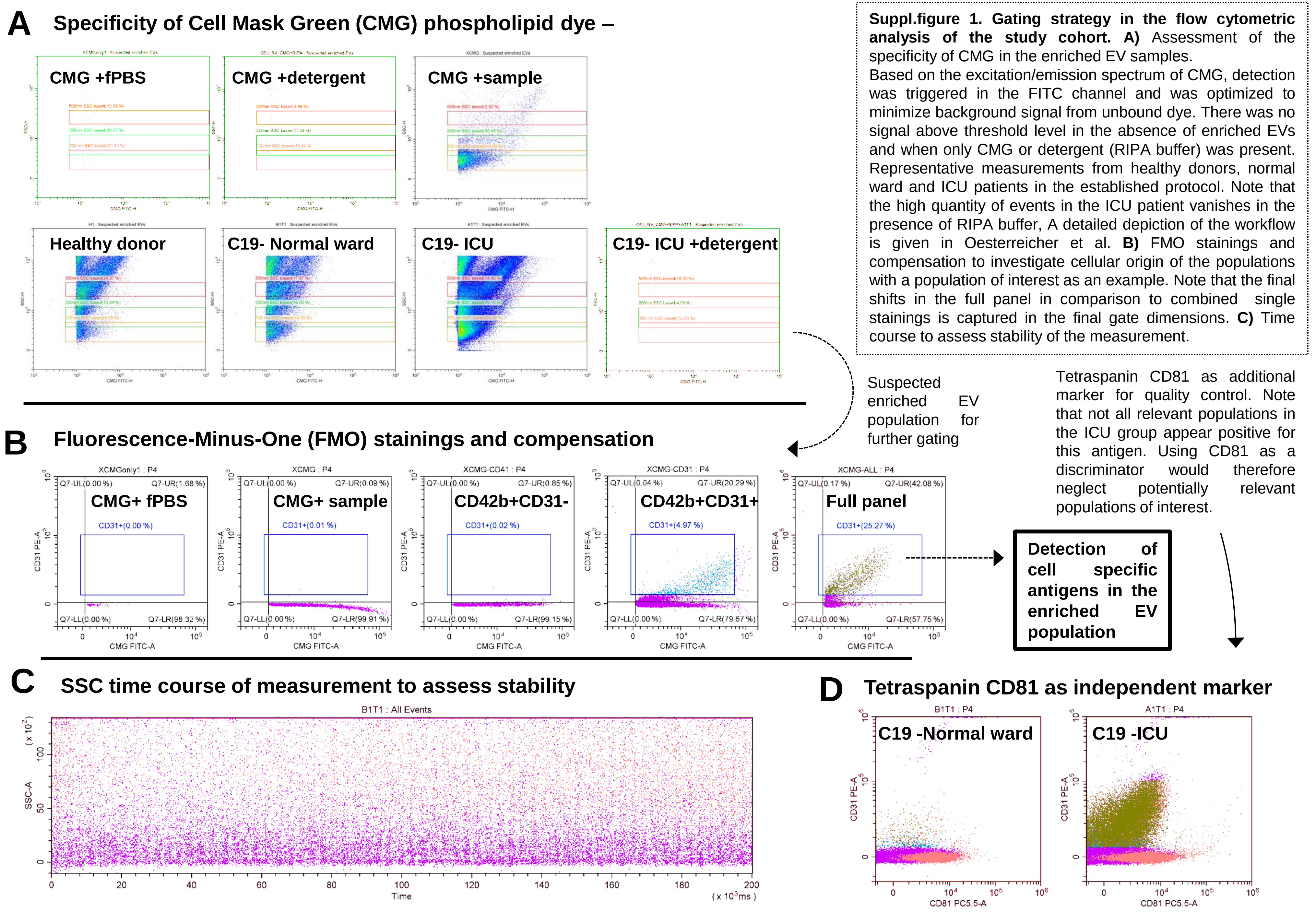

Suppl.figure 1. Gating strategy in the flow cytometric analysis of the study cohort. A) Assessment of the specificity of CMG in the enriched EV samples.
Based on the excitation/emission spectrum of CMG, detection was triggered in the FITC channel and was optimized to minimize background signal from unbound dye. There was no signal above threshold level in the absence of enriched EVs and when only CMG or detergent (RIPA buffer) was present. Representative measurements from healthy donors, normal ward and ICU patients in the established protocol. Note that the high quantity of events in the ICU patient vanishes in the presence of RIPA buffer, A detailed depiction of the workflow is given in Oesterreicher et al. **B)** FMO stainings and compensation to investigate cellular origin of the populations with a population of interest as an example. Note that the final shifts in the full panel in comparison to combined single stainings is captured in the final gate dimensions. **C)** Time course to assess stability of the measurement.
